# Supplementary material for: Optimizing type, date, and dose of compost fertilization of organic cotton under climate change in Mali: A modeling study
Source: PLoS One. 2024 Aug 14;19(8):e0308736. doi: 10.1371/journal.pone.0308736 (PMC11324123; doi:10.1371/journal.pone.0308736)
Supplement: Attachment — Submitted filename: Review sheet_PlosONE.docx [file pone.0308736.s002.docx]

General comments:

| **Line number** | **Comments** |
| --- | --- |
| 32, 33 | Key words are lengthy, Try to reduce it and give those key words that best describe the article |
| 85 | Check the journal guidelines. It should be Fig 1 not Figure 1 |
| 87 | Check the figure caption style and follow journal guideline |
| 186 | Check the journal guidelines. It should be Fig 2 not Figure 2 |
| 190 | Check the figure caption style and follow journal guideline |
| 214 | Check the abbreviation and use same abbreviation uniformly throughout the manuscript |
| 235 | Interchange the minimum and maximum words according to the data |
| 242 | Check the solar radiation data, use point instead of comma |
| 254 | Check the journal guidelines. It should be Fig 3 not Figure 3 |
| 258 | Use past tense |
| 268 | Check the figure caption style and follow journal guideline |
| 274 | See the citation format |
| 282 | Modify figure citation according to the journal guideline |
| 296 | Figure caption |

**Specific comments**:

1. Line no 64-78: In this paragraph, authors should highlight the urgency or potential lacunas that this study addresses. The title highlights the type, date and dose of organic input, in that direction, authors should highlight that how these factors are currently affecting the seed yield of cotton in Mali with references. The gaps and objectives of this study has to come in this section.

**Verdict:**

The DSSAT model is an important tool to simulate the future climate change scenarios to see the effect of our management on crop growth and suitably modify or adapt a new strategy for best outputs and benefits. This study has comprehensively highlighted the date, dose and type of organic manure that can be adopted by Mali cotton farmers keeping the climate change scenario in mind to get an upper hand in getting higher yield. The manuscript is very well written and experimental methods as well as the model optimization are well executed. Very minor rectifications are required which are highlighted in the comment section. The study should be published with having those rectification incorporated.
