# Supplementary material for: Optimizing type, date, and dose of compost fertilization of organic cotton under climate change in Mali: A modeling study
Source: PLoS One. 2024 Aug 14;19(8):e0308736. doi: 10.1371/journal.pone.0308736 (PMC11324123; doi:10.1371/journal.pone.0308736)
Supplement: Attachment — Submitted filename: Review report.docx [file pone.0308736.s003.docx]

Composting is an effective and sustainable technique in organic farming because it helps to reduce nutrient loss through leaching and reduces the need for synthetic fertilizers. It acts as a natural storehouse of both micronutrients like zinc, iron, copper, and manganese, and macronutrients such as nitrogen, phosphorus, potassium, calcium, and magnesium etc. The specific nutrients present depend on the type and composition of the organic material being composted. Additionally, composting provides a reliable method for improving the physical, chemical, and biological qualities of soil in agricultural contexts. Furthermore, applying compost leads to higher crop yields, mainly because it enhances soil fertility and provides essential micronutrients and macronutrients. Numerous studies have highlighted the beneficial effects of compost on the growth and physical characteristics of cotton plants.

Thus, before accepting needs to be modify as per the comments given below:

Some of the study reported that Cotton is C3 and CO_2_ enrichment enhances yield (Sankaranarayanan et al., 2010) thus specifying the date application of compost enhanced the yield of cotton crop need to be more discussed.

In Introduction, importance of the study needs to be highlighted with more recent references so that study can be justified. Write the importance of this study in global context.

Why DSSATCSM-CROPGRO-Cotton model was used, need more justification as well as include R^2^ value in both the RCPs. Is this model considered mineralization of compost in soil for prediction?

Whether different sources of composts (elemental composition) were analyzed or not?

In this study, date of sowing of cotton is July 01, 2021, Generally, application of compost 2-3 week before sowing is recommended, but in this study application in mid-May emphasized therefore proper discussion is required indicating to show why application of this model is important rather than going for general recommendation
