# Supplementary material for: Optimizing type, date, and dose of compost fertilization of organic cotton under climate change in Mali: A modeling study
Source: PLoS One. 2024 Aug 14;19(8):e0308736. doi: 10.1371/journal.pone.0308736 (PMC11324123; doi:10.1371/journal.pone.0308736)
Supplement: Attachment — Submitted filename: Response to Reviewers.docx [file pone.0308736.s004.docx]

The authors are grateful to the reviewers for their comments, which have helped improve the manuscript. In the revised manuscript with track changes, revisions related to reviewer #1's comments are highlighted in yellow, and those of reviewer #2 are highlighted in blue.

Reviewer #1:

C1. Line no 64-78: In this paragraph, authors should highlight the urgency or potential lacunas that this study addresses. The title highlights the type, date and dose of organic input, in that direction, authors should highlight that how these factors are currently affecting the seed yield of cotton in Mali with references. The gaps and objectives of this study has to come in this section.

R1. Thank you. The introduction has been augmented to present better the gap in knowledge and the study's objective.

C2. L32-33. Key words are lengthy. Try to reduce it and give those key words that best describe the article

R2. We modified the keywords, making them shorter and ensuring they describe the content of the article.

C3. L85. Check the journal guidelines. It should be Fig 1 not Figure 1

R3. Thank you. All references to figures have been updated to comply with the journal guidelines.

C4. L87. Check the figure caption style and follow journal guideline

R4. Thank you. All figure captions have been updated to comply with the journal guidelines.

C5. L186. Check the journal guidelines. It should be Fig 2 not Figure 2

R5. Thank you. All references to figures have been updated to comply with the journal guidelines.

C6. L190. Check the figure caption style and follow journal guideline

R6. Thank you. All figure captions have been updated to comply with the journal guidelines.

C7. L214. Check the abbreviation and use same abbreviation uniformly throughout the manuscript

R7. We replaced NMRSE by nRMSE

C8. L235. Interchange the minimum and maximum words according to the data

R8. We interchanged the min and max to correspond to what they refer to in the text.

C9. L242. Check the solar radiation data, use point instead of comma

R9. We replaced the comma by point.

C10. L254. Check the journal guidelines. It should be Fig 3 not Figure 3

R10. Thank you. All references to figures have been updated to comply with the journal guidelines.

C11. L258. Use past tense.

R11. We used past tense (had instead of has).

C12. L268. Check the figure caption style and follow journal guideline.

R12. Thank you. All figure captions have been updated to comply with the journal guidelines.

C13. L274. See the citation format.

R13. Thank you, all references to figures have been updated to comply with the journal guidelines.

C14. L282. Modify figure citation according to the journal guideline

R14. Thank you. All references to figures have been updated to comply with the journal guidelines.

C15. L296. Figure caption.

R15. Thank you. All figure captions have been updated to comply with the journal guidelines.

Reviewer #2:

C16. Some of the study reported that Cotton is C3 and CO2 enrichment enhances yield (Sankaranarayanan et al., 2010) thus specifying the date application of compost enhanced the yield of cotton crop need to be more discussed.

R16. Thank you for your comment. We indeed used the suggested review paper in our introduction to highlight the importance of organic manure like compost in increasing soil organic content in cotton systems under climate change to reduce the impact of climate change.

C17. In introduction, importance of the study needs to be highlighted with more recent references so that study can be justified. Write the importance of this study in global context.

R17. Thank you very much; please kindly see the response to comment C1 in yellow in the introduction. Some recent publications have been added (2 from 2022, 1 from 2023). We rewrote the end of the introduction to highlight better the importance of this study in the global context.

C18. Why DSSAT CSM-CROPGRO-Cotton model was used, need more justification as well as include R2 value in both the RCPs. Is this model considered mineralization of compost in soil for prediction?

R18. Thank you for your comment. We justified the choice of model and described it better. The details are highlighted in blue in the M&M section 2.3.1. Under both RCPs, this is an in-silico experiment and no observation can be compared to simulated future performance under climate change conditions. Under such conditions, no R2 can be computed.

C19. Whether different sources of composts (elemental composition) were analyzed or not?

R19. The types of composts were analyzed for elemental composition, a table was added (Table 2).

C20. In this study, date of sowing of cotton is July 01, 2021, Generally, application of compost 2-3 week before sowing is recommended, but in this study application in mid-May emphasized therefore proper discussion is required indicating to show why application of this model is important rather than going for general recommendation

R20. Thank you for your comment. The reference 28 specifies that organic manure is usually applied between March and April in Mali. In addition, an unpublished survey of farmers producing organic cotton in Mali has identified that compost is also commonly applied in March or April but rarely after. Our work highlights the need for a change of practice.
