# Supplementary material for: Optimizing type, date, and dose of compost fertilization of organic cotton under climate change in Mali: A modeling study
Source: PLoS One. 2024 Aug 14;19(8):e0308736. doi: 10.1371/journal.pone.0308736 (PMC11324123; doi:10.1371/journal.pone.0308736)
Supplement: Attachment — Submitted filename: Reviewer sheet_PONE-D-23-33999_R1.docx [file pone.0308736.s005.docx]

**General comments:**

General comments they have worked on which is fin but still the research gap part is lacking. They have added only a paragraph that to not ensuring why application of compost and their timing of application is important for Mali cotton farmers. I understand that application of manure organics undoubtedly improves the soil and crop performance. But it has to come in the manuscript. Without highlighting any potential gap or requirement of this research how we are going to justify that our work is the need of the hour. I request authors to include these things. What is the objective here. What they are trying to address by optimizing the date dose and timing of manure application and how it is going to address the climate change issues. Ultimately, if they address these things then it would be good.

And again, regarding publication, I think ultimately, it’s the prerogative of the editor to decide if they want to go for publication or not. Thank you.
